# Widespread Lewy body and tau accumulation in childhood and adult onset dystonia-parkinsonism cases with *PLA2G6* mutations

**DOI:** 10.1016/j.neurobiolaging.2010.05.009

**Published:** 2012-04

**Authors:** Coro Paisán-Ruiz, Abi Li, Susanne A. Schneider, Janice L. Holton, Robert Johnson, Desmond Kidd, Jeremy Chataway, Kailash P. Bhatia, Andrew J. Lees, John Hardy, Tamas Revesz, Henry Houlden

**Affiliations:** a Department of Molecular Neuroscience, UCL Institute of Neurology, London, UK; b Queen Square Brain Bank, UCL Institute of Neurology, London, UK; c Sobell Department of Motor Neuroscience and Movement Disorders, UCL Institute of Neurology, London, UK; d Rita Lila Weston Institute of Neurological Studies, London, UK; e Department of Pediatrics, University of Maryland, Baltimore, MD, UK; f Department of Clinical Neurosciences, Royal Free and University College Medical School, London, UK

**Keywords:** *PLA2G6*, *PANK2*, Lewy bodies, tau, parkinsonism

## Abstract

The 2 major types of neurodegeneration with brain iron accumulation (NBIA) are the pantothenate kinase type 2 (*PANK2*)-associated neurodegeneration (PKAN) and NBIA2 or infantile neuroaxonal dystrophy (INAD) due to mutations in the phospholipase A2, group VI (*PLA2G6*) gene. We have recently demonstrated clinical heterogeneity in patients with mutations in the *PLA2G6* gene by identifying a poorly defined subgroup of patients who present late with dystonia and parkinsonism. We report the clinical and genetic features of 7 cases with *PLA2G6* mutations. Brain was available in 5 cases with an age of death ranging from 8 to 36 years and showed widespread alpha-synuclein-positive Lewy pathology, which was particularly severe in the neocortex, indicating that the Lewy pathology spread corresponded to Braak stage 6 and was that of the “diffuse neocortical type”. In 3 cases there was hyperphosphorylated tau accumulation in both cellular processes as threads and neuronal perikarya as pretangles and neurofibrillary tangles. Later onset cases tended to have less tau involvement but still severe alpha-synuclein pathology. The clinical and neuropathological features clearly represent a link between *PLA2G6* and parkinsonian disorders.

## 1 Introduction

In the early part of the twentieth century the work of Seitelberger (Hallervorden and Spatz, 1922; Seitelberger, 1971) and others described the clinicopathological features of the 2 commonest forms of neurodegeneration with iron deposition, that is neuroaxonal dystrophy (NAD) and Hallervorden-Spatz disease, now renamed neurodegeneration with brain iron accumulation (NBIA) (Gregory et al., 2009).

The 2 major types of NBIA are recessively inherited disorders, NBIA type 1 is due to mutations in the pantothenate kinase 2 gene (*PANK2*) and often called pantothenate kinase associated neurodegeneration (PKAN) (Hayflick et al., 2003; Zhou et al., 2001) and NBIA type 2 or infantile neuroaxonal dystrophy (INAD) (previously Seitelberger's disease) is due to mutations in the phospholipase A2, group VI (*PLA2G6*) gene (Khateeb et al., 2006; Morgan et al., 2006; Seitelberger, 1971). According to the age of onset and progression, NAD can be separated into: (1) infantile or classical type (INAD), and (2) juvenile/adult onset or atypical NAD. A further group comprises idiopathic NBIA where there is brain iron on imaging but no genetic abnormality in the *PLA2G6* and *PANK2* genes (Gregory et al., 2009; Morgan et al., 2006). Two other genes also cause NBIA, acoeruloplasminaemia due to mutations in the (ceruloplasmin) *CP* gene (Morita et al., 1992) and neuroferritinopathy, caused by mutations in the *FTL* (ferritin light polypeptide) gene. Patients with mutation of these 2 genes usually present with an adult onset movement disorder (Curtis et al., 2001). The vast majority of PKAN cases have imaging evidence of high iron accumulation but this is only present in half the cases with *PLA2G6* mutations. PKAN accounts for approximately 50% of the cases with NBIA (Hayflick et al., 2006; Khateeb et al., 2006; Morgan et al., 2006). Recently families with adult onset dystonia-parkinsonism were found to have mutations in the *PLA2G6* gene but absent iron deposition on magnetic resonance imaging (MRI) (Paisan-Ruiz et al., 2009; Sina et al., 2009).

The clinical phenotype of NBIA is broad, although there are some characteristic features (Gregory et al., 2009; Hayflick et al., 2003). Most cases present before the age of 5 years with developmental delay, dystonia, rigidity, dysarthria, and ataxia. Onset between 2 and 18 years is characteristic for the juvenile type and onset after 18 years for the adult type or atypical NAD. The finding of patients with *PLA2G6* mutations and L-dopa responsive adult onset dystonia-parkinsonism adds a later onset subgroup to the NBIA clinical spectrum. MRI has been of great importance in distinguishing the clinical and genetic forms of NBIA. In cases with PKAN mutations a region of hyperintensity (necrosis or edema) in the globus pallidus is seen with surrounding hypodensity (region of high iron) on T2-weighted images (Hayflick et al., 2006). This “eye of the tiger” sign is associated with mutations in the *PANK2* gene (Arawaka et al., 1998; Barbosa et al., 1995; Galvin et al., 2000; Guillerman, 2000; Hajek et al., 2005; Hayflick and Westaway, 2006; Hermann et al., 2000) but is not pathognomonic (McNeill et al., 2008). In classical INAD, abnormal iron mainly accumulates in the globus pallidus and sometimes in the substantia nigra in the more atypical cases (Gregory et al., 2009; Hayflick et al., 2003).

In the past it has been possible to differentiate NAD from PKAN pathologically mainly by the distribution of dystrophic neuroaxonal swellings (spheroids). These have been shown by immunohistochemistry to be immunoreactive for neurofilament, amyloid precursor protein, ubiquitin, and alpha-synuclein (Arawaka et al., 1998; Galvin et al., 2000; Gregory et al., 2009; Neumann et al., 2000; Newell et al., 1999; Odawara et al., 1992; Saito et al., 2000; Sugiyama et al., 1993; Tofaris et al., 2007; Tuite et al., 1996; Wakabayashi et al., 1999), although larger spheroids may be negative for neurofilament. However, with identification of the *PLA2G6* gene, it has become clear that there is also pathological heterogeneity in NAD, as cases with clinical and pathological features of INAD were found to be negative for *PLA2G6* mutations and patients with *PLA2G6* mutations have been identified without axonal spheroids (Gregory et al., 2009). The neuropathological examination of only 1 case with a confirmed *PLA2G6* mutation has been reported in a patient with atypical neuroaxonal dystrophy and an age of onset of 3 years. This case had typical axonal spheroids, iron deposition, Lewy bodies, and Lewy neurites in the substantia nigra and cortex as well as tau immunoreactive tangles (Gregory et al., 2009).

To expand the neuropathological spectrum of neuroaxonal dystrophy caused by *PLA2G6* mutations and investigate the overlap with other parkinsonian disorders, we identified 7 genetically-proven cases with infantile through to adult onset disease and report the clinical and neuropathological features.

## 2 Methods

This project was approved by the Joint Local Research Ethics Committee of the National Hospital for Neurology and Neurosurgery. Brains are stored in the Queen Square Brain Bank, London (QSBB) and the Brain and Tissue Banks for Developmental Disorders, Baltimore (BTBDD) by obtaining appropriate consents.

### 2.1 Patients

The clinical features of the patients examined are described in the results section and Table 1. Cases 1 and 2 were from the QSBB. Cases 3–6 were from the National Institute of Child Health and Human Development (NICHD), Brain and Tissue Bank for Developmental Disorder. Tissue from these disorders is rare as brain donation is not frequently made by families; we contacted as many brain banks as possible to obtain tissue from cases with a young onset complex dystonia or parkinsonian disorder. In cases 1, 2, and 4 detailed clinical notes were available, clinical details in cases 3 and 5 were minimal. Cases 1, 3, 4, and 5 underwent detailed neuropathological examination. Tissue from a cortical biopsy was available in case 2. The neuropathological findings of case 2 (Tofaris et al., 2007) and case 5 (Galvin et al., 2000) had been reported earlier. Two further cases (case 6 and 7) only had a sural nerve biopsy carried out.

### 2.2 Genetics

DNA was extracted from brain tissue or blood using a standard phenol chloroform method. All exons of the *PANK2* gene were sequenced as was exon 4 of the ferritin light chain gene (the only exon shown to have mutations) (Curtis et al., 2001) as both genes have an NBIA phenotype and were found to be negative.

All 16 coding exons of the *PLA2G6* gene (reference sequence NM_003560) were sequenced, as with the other genes given above, using standard Sanger PCR sequencing (Roche Faststart Mastermix, Roche, Indianapolis), purification (Millipore, Billerica, MA), Bigdye (ABI/Perkin Elmer, Waltham, MA) sequencing reaction, Bigdye clean up (ABgene, Epsom, UK) and running on an ABI3730XL sequencer. Primers were designed to amplify each gene exon and flanking 50 bp intronic sequences (Table 1). The controls screened were from the UK Wellcome trust 1958 birth cohort and the *Centre d'Etude du Polymorphisme Humain* (CEPH) Human Genome Diversity Panel (HGDP), these samples are available from the Wellcome trust or CEPH. Mutations were numbered from the ATG start site of the *PLA2G6* gene.

### 2.3 Neuropathology

The entire brain was examined in case 1; paraffin blocks from representative brain areas were available from case 4. In case 2 frontal cortical biopsy tissue was available and frozen material was only available from case 3. Seven μm thick tissue sections were stained with hematoxylin and eosin, Luxol fast blue cresyl violet, Perl's stain for iron and Gallyas silver methods. Immunohistochemistry was performed with antibodies to glial fibrillary acidic protein (Dako, 1:1000, Ely, UK), alpha-synuclein (Novocastra, 1:50, Newcastle, UK), phospho-alpha-synuclein (Abcam,1:1000, Cambridge, UK), tau (AT8, Autogen Bioclear, 1:500, Nottingham, UK), AT100, Autogen Bioclear 1:200, Nottingham, UK), ubiquitin (Dako, 1:200, Ely, UK), p62 (BD Transduction, 1:100, Oxford, UK, neurofilament (RT97 Novocastra, 1:50, Newcastle, UK), SMI31 Sternberger Monoclonals, 1:5000, Cambridge, UK), anti-mouse or anti-rabbit secondary antibody (Dako, Ely, UK) was used as appropriate, followed by incubation with ABC (Vector, Peterborough, UK). Color was developed by diaminobenzidine/H_2_O_2_. Sections were counterstained with Mayer's hematoxylin.

## 3 Results

Clinical details are summarized in Table 2. Cases 1 and 2 were from the host brain bank where complete clinical details were available, cases 3–7 were from another brain bank where copies of the clinical notes were obtained but these cases had not been seen by any of the coauthors. Cases 3, 5, 6, and 7 all had the typical clinical features of classical NAD with psychomotor regression at around 1 year of age and progressive loss of ambulation before aged 5 years. All cases had progressive cognitive and developmental deterioration, with ataxia, speech, and swallowing problems. Visual problems occurred in case 3 (bilateral optic atrophy) and case 6, bilateral hearing loss, seizures, flaccidity, and muscle cramps in case 5. Imaging demonstrated cerebellar atrophy in all cases and iron accumulation only in case 6 in the basal ganglia region, typical of NAD, although imaging was done early on in the disease in cases 3 and 5. Case 4 had juvenile or atypical NAD with an age of onset in childhood with dystonia and paroxysmal jerky body movements that were consistent with myoclonic jerks; an electroencephalogram (EEG) showed right-sided spike and slow wave discharges. Although this patient improved with feeding and weight gain after a gastrostomy tube was inserted, he continued to globally progress and died aged 18 years. The case of death in all cases was aspiration or ventilation-related pneumonia.

Cases 1 and 2 had a later age of onset, slower disease progression, and both developed parkinsonian features. Although the overall clinical features of these 2 cases were different than idiopathic Parkinson's disease (PD) there is overlap and both cases are discussed in detail below and given in Table 2. Case 1 had a diagnosis of “adult-onset progressive ataxia, spasticity, and parkinsonism”. She had no relevant past medical or psychiatric history apart from toe walking. At the age of 18 she had increasing problems with overt urinary incontinence and constipation. She was 28 years old when presented to a neurologist. Her examination revealed normal eye movements, mild cerebellar dysarthria, and ataxia, and mild pyramidal weakness in all limbs. Brain imaging and central and peripheral electrophysiology, cerebrospinal fluid (CSF), and muscle biopsy were all normal. Neuropsychometry suggested mild cognitive decline. These problems progressed, she could no longer work from age 30 and became wheelchair-bound due to increasing ataxia and spasticity at the age of 32 years. She developed psychiatric features with aggression, swallowing difficulties, and dysarthria and a sensory neuropathy. Parkinsonism with rigidity and bradykinesia was first noted at age 34; this involved the upper limbs and affected the gait in a symmetrical pattern. Dystonia was absent. Repeat MRI showed cerebellar atrophy. She further progressed, became bedbound with severe akinetic rigidity, was mute, and unable to communicate. She died of bronchopneumonia at the age of 36.

Case 2 had a diagnosis of an adult-onset progressive dystonia-parkinsonism disorder. She was well until the age of 22 years when she developed psychiatric features with mood changes, irritability, aggression, and transient visual hallucinations. She had a rapidly progressive decline in language abilities and became mute within 9 months. She also had weight loss and feeding problems. She received psychiatric treatment including electroconvulsive therapy and haloperidol, which resulted in extrapyramidal side effects. These improved rapidly with clozapine as did her mood. At the age of 24, her extrapyramidal features returned and she developed progressive tremor, bradykinesia, and micrographia with a shuffling gait and falls. L-dopa therapy was initially successful but later on in the disease course she did not respond and developed limb and orofacial dyskinesias a short time after dosing. On examination at age 28, her higher mental functions were severely impaired on verbal and nonverbal tests of reasoning. She was hypomimic with reduced visual perceptual and visuospatial skills. Pursuit eye movements were jerky, and saccades were slow. She had marked dystonic posturing of all extremities. Her reflexes were brisk and the plantar responses were spontaneously extensor. Brain MRI at age 27 years showed severe cerebral, predominantly frontotemporal atrophy. Low signal intensity, indicative of abnormal iron deposition in the globus pallidus, cerebral peduncles, and substantia nigra without “eye of the tiger” sign were present on T2-weighted images. This patient was investigated in detail for other acquired caused of dystonia and parkinsonism; these were normal and included metabolic screening, muscle, rectal biopsy, and bone marrow biopsies, genetic testing for SCA1–3, 6, 7, 12, 17, Huntington disease, dystonia 1 gene (*DYT1*)-related primary dystonia, alpha-synuclein and dentatorubropallidoluysian atrophy (DRPLA). After these investigations proved negative a full thickness right frontal brain biopsy was carried out; these results are discussed below.

### 3.1 Mutation analysis

Sequencing of the *PLA2G6* gene revealed the presence of mutations in the examined cases (Tables 2 and 3). Ten different *PLA2G6* mutations were identified and confirmed by repeat sequencing. Six of them, including frameshift, missense, splice site, and stop mutations were novel (Table 2); conserved in species and not identified in controls. The 2 novel missense mutations, *p*.*R600Q* and *p*.*T572I*, were also not identified in 548 and 380 control chromosomes, respectively, and both were conserved across species. Cases 2 and 6 were homozygous mutations, cases 1, 3, and 5 were compound heterozygous. Cases 4 and 7 had single heterozygous mutations. Case 7 was clinically diagnosed with peripheral nerve pathology. In this case a single heterozygous mutation was identified in the *PLA2G6* gene. In cases 4 and 7 we analyzed the DNA on Human610-Quad-Infinium HD BeadChips to investigate the *PLA2G6* gene for deletions. However, this array platform only contains 14 markers located in exon 2 and introns 1, 2, 3, 4, 5, 8, and 10 at the *PLA2G6* locus; thus, we could have missed a small heterozygous deletion affecting only a small part of the gene. The fact that both mutations are found in heterozygous states also suggests that if there are deletions at the *PLA2G6* locus in these cases, these will not affect the entire locus. Hence, the *PLA2G6* deletions in these cases are expected to be small and thus not appreciable through the single nucleotide polymorphism (SNP) genotyping assays. Case 7 was homozygous for the 14 single nucleotide polymorphisms at the *PLA2G6* locus, suggesting the presence of a small deletion on 1 allele (likely compound heterozygous nonsense mutation/deletion). Case 4 was homozygous in several regions of the gene with a deletion possible of exons 2, 5 to 8, or 10 to 17 (Supplementary Material). Because of the single heterozygous changes identified in cases 4 and 7 and the lack of absolute proof of a second mutation these 2 cases should be considered only probable *PLA2G6* NBIA.

### 3.2 Neuropathology

**Fig. 1:**
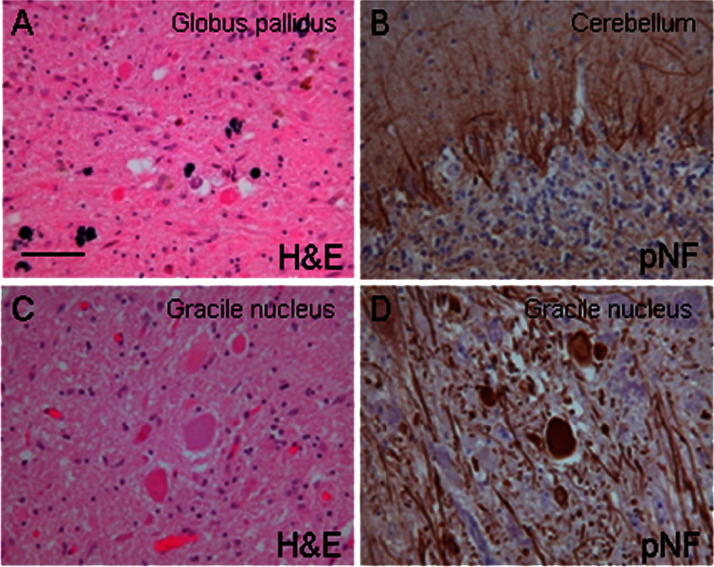
(A) Significant degree of pigment deposition in the globus pallidus in case 4. (B) Empty baskets highlighting significant Purkinje cell loss in the cerebellar cortex in case 1. (C) Large neuroaxonal swellings in the gracile nucleus in case 1, which were often immunoreactive for neurofilament (D). (A and C) Hematoxylin and eosin (H&E); (B and D) phospho-neurofilament immunohistochemistry (pNF) (RT97 antibody). The bar on (A) represents 40 μm.

**Fig. 2:**
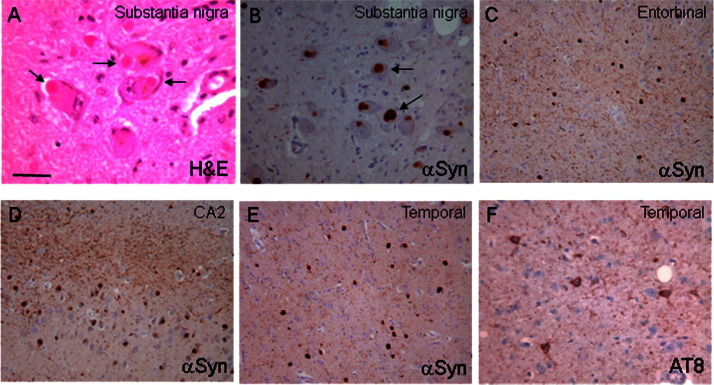
(A and B) Frequent Lewy bodies in substantia nigra neurons in case 1. Severe Lewy pathology is demonstrated in the entorhinal cortex (C), CA2 hippocampal subregion (D), and temporal neocortex (E) in case 1. The tau pathology was extensive in case 3 and demonstrated here in the temporal cortex. (A) Hematoxylin and eosin (H&E); (B–E) alpha-synuclein immunohistochemistry (αSyn); (F) tau immunohistochemistry (AT8 antibody). The bar on (A) represents 80 μm.

The neuropathological data are summarized in Table 4. Gross inspection revealed cerebral atrophy in case 5 and cerebellar cortical atrophy was documented in cases 1 and 5. A rusty discoloration of the globus pallidus was found in case 4. Severe pallor of the substantia nigra was seen in case 1 (age at death 36 years). On microscopic investigation the globus pallidus contained small clusters of iron-laden macrophages in case 1, while the iron deposition was severe in case 4 (Fig. 1A) and widespread in case 5. There were numerous axonal swellings in case 1, 3, 4, and 5 (Fig. 1B and C) in the basal ganglia and brainstem. In case 1 and 5 where the spinal cord was available, there were also numerous axonal spheroids in the cord. On hematoxylin and eosin stained histological sections the axonal swellings appeared as large spherical structures, up to 100 μm in diameter and were mostly stained with the antineurofilament antibodies. There was variable depletion of cerebellar cortical neurons (granular cells more than Purkinje cells) accompanied by marked astrocytosis in cases 1, 4, and 5 (Fig. 1D), in which the cerebellum was available for assessment. The substantia nigra neurons were depleted in case 1. In this case and also in case 4 Lewy bodies were readily found in the nigra on the hematoxylin and eosin- stained sections. Alpha-synuclein positive Lewy bodies were widespread in cases 1, 4 and 5. In case 2, where only the biopsy specimen of the frontal cortex was available, Lewy bodies were frequent. Lewy bodies were restricted to the medulla in case 3, corresponding to Braak stage 1. In cases 1 and 4 the morphological appearances and the topographical distribution of the Lewy body type pathology were comparable to those seen in severe, end-stage Parkinson's disease and cases with dementia with Lewy bodies (Halliday et al., 2008; McKeith et al., 2005). Accordingly, severe Lewy body pathology was observed in the “dementia with Lewy bodies” consensus areas, including cerebral cortex, basal forebrain, hippocampal formation, and brainstem nuclei corresponding to “diffuse neocortical Lewy body type pathology” (McKeith et al., 2005) and Braak stage 6 (Braak et al., 2003) in both case 1 and 4 (Fig. 2A–E). Lewy bodies and Lewy neurites in nigra and/or cortex were ubiquitin-positive and were confirmed to contain alpha-synuclein phosphorylated at Serine 129. In addition, alpha-synuclein accumulation was observed in axonal swellings in all postmortem cases (cases 1, and 3–5).

There was extensive tau pathology with neurofibrillary tangles, pretangles, and neuropil threads in case 4 corresponding to Braak and Braak stage V (Fig. 2F). The AT8-positive structures were also AT100-positive and Gallyas silver-positive, which is indicative of tau filaments. Tau-positive threads and neurofibrillary tangles were restricted to the transentorhinal cortex in case 1 (Braak and Braak stage I). There were numerous, tau-positive neuropil threads in case 2 investigated by a frontal cortical biopsy and tau-positive glia in hippocampus and entorhinal cortex were described in case 5 (Galvin et al., 2000).

## 4 Discussion

We describe the genetic and clinical features of a total of 7 cases with NAD associated with *PLA2G6* mutations. An important addition to the literature provided by this study is the neuropathological data on cases with varying ages of onset, including 2 adult onset cases. A wide distribution of missense, nonsense, and frameshift mutations was identified in the *PLA2G6* gene, which were not identified in controls. The clinical features in the classical INAD and juvenile cases were very similar and have been previously well described. Cases 1 and 2 presented later with ages of onset of 18 and 22; they had a slower progression with prominent psychiatric symptoms: rigidity, dystonia, and parkinsonian features. Case 2 responded to L-dopa treatment but was very sensitive, developing the side effects of worsening dystonia and dyskinesias. L-dopa was not prescribed in case 1.

Neuroaxonal dystrophy and alpha-synuclein pathology with Lewy bodies and Lewy neurites was seen in all cases, in which the brain was examined postmortem and severe alpha-synuclein pathology was also confirmed in the case investigated by a cerebral cortical biopsy. Tau pathology was also present in 3 postmortem cases (cases 1, 4, and 5) and the biopsy case (case 2). In cases 1 and 4 the Lewy body pathology was severe in the neocortex and also in the basal forebrain, hippocampal formation, and brainstem nuclei corresponding to Braak stage 6 (Braak et al., 2003) and to “diffuse neocortical Lewy body type pathology” according to the consensus criteria established for the diagnosis of dementia with Lewy bodies (McKeith et al., 2005).

In keeping with previous observations (Gregory et al., 2008) a striking aspect of our cohort of NBIA type 2 cases with *PLA2G6* mutations and consisting of young individuals (age at death ranging between 8 and 36 years), is the presence of alpha-synuclein pathology, which was severe in 3 cases and in 2 of the postmortem cases it corresponded to that seen in end stage Parkinson's disease and dementia with Lewy bodies (Halliday et al., 2008; McKeith et al., 2005). The widespread distribution of Lewy bodies in the brains in our patients may explain the psychiatric features (in cases 1 and 2) and psychomotor retardation in other cases. The Lewy bodies in the cases reported here with L-dopa responsive parkinsonian features and the reported cases with dystonia and parkinsonism clearly represent a link between cases with *PLA2G6* mutations and Parkinson's disease. Our observations are also in line with recent reports of PLA2G6 null mice showing accumulation of alpha-synuclein in axonal and cytoplasmic aggregates (Malik et al., 2008).

In 4 of the cases there was also hyperphosphorylated tau accumulation in both neuronal processes (threads) and perikarya (pretangles and neurofibrillary tangles) in cases 1 and 4 and in threads in case 2. The tau pathology was subtle and mostly restricted to the entorhinal cortex in case 1, but was severe in both mediotemporal structures and frontal and temporal neocortex in case 4 corresponding to Braak stage V. In case 2 with disease onset in early adulthood numerous, tau-positive fine threads were present in the frontal cortical biopsy specimen. The co-occurence of hyperphosphorylated tau deposition in these cases is of considerable interest as the young age of the patients excludes an “age-related, incidental” phenomenon and amyloid-β plaques were not seen in any of the cases. Codeposition of tau and alpha-synuclein has been documented in both familial PD (Duda et al., 2002) and sporadic PD (Ishizawa et al., 2003; Neumann et al., 2009). The extent and severity of the tau pathology in some of our cases and in a previously reported case with *PLA2G6* mutation (Gregory et al., 2008) also raise the possibility of a mechanistic link not only between *PLA2G6* mutations and alpha-synuclein accumulation, but also between *PLA2G6* mutations and tau hyperphosphorylation and deposition.

The cases reported here all had the neuropathological features of neuroaxonal dystrophy, alpha-synuclein-positive Lewy bodies and Lewy neurites. In all but one case, tau pathology was also present. The clinical features of the cases reported are different. There is a possible genotype phenotype correlation that may explain the differences between the classical INAD (childhood) and the late-onset atypical NAD cases, although the dataset here is small. In the classical INAD cases the mutation distribution occurs throughout the gene with nonsense and frameshift mutations occurring more frequently; these mutations are likely to cause loss of the PLA2G6 functional protein. Atypical cases have a greater number of missense and compound heterozygous mutations toward the 3′ prime end of the gene and this is particularly the case in the 4 families with adult atypical NAD and parkinsonism (Table 5). Families 1, 2, and the Iranian family were previously reported (Paisan-Ruiz et al., 2009; Sina et al., 2009). The *PLA2G6* mutation position and type may give some indication of the possible phenotype, but this is not comprehensive. Gregory and colleagues recently described similar *PLA2G6* mutations causing a classical INAD phenotype and a codon 741 mutation (Gregory et al., 2009).

This report further defines the clinical features and neuropathology of PLA2G6 related NAD. We particularly highlight the role of PLA2G6-related neurodegeneration in patients with juvenile and adult-onset disease complicated by dystonia and parkinsonism with normal MRI imaging. The finding of Lewy body pathology in PLA2G6 bridges a link with other neurodegenerative diseases like Parkinson's disease and dementia with Lewy bodies and may shed light on shared pathological pathways.

## Disclosure statement

None of the authors has stated any conflict of interest.

This project was approved by the Joint Local Research Ethics Committee of the National Hospital for Neurology and Neurosurgery. Brains are stored in the Queen Square Brain Bank, London (QSBB) and the Brain and Tissue Banks for Developmental Disorders, Baltimore (BTBDD) by obtaining appropriate consents.

## Figures and Tables

**Table 1:** PCR and sequencing primers used to amplify *PLA2G6*, *PANK2* and *FTL*

	Forward sequence (5′ to 3′)	Reverse sequence (5′ to 3′)
*PLA2G6*		
Exon 2	GTGTCTGTGCAGGAAACCG	GCCAATAAGACCTCCAATCC
Exon 3	TGATTCCAGCAGGGATGTG	AACTATGGAGGGGAACCGAG
Exon 4	AAAGTCCGAGTTTCCGAGTG	AGGCCTGAGAGTGACACCTG
Exon 5	GTGATCCACCCACCTTGG	TGGTGGATACTGCTTGCCTC
Exon 6	CTTCATCCCACGCCACG	GAACCTGCTTCCTGAGGG
Exon 7	TCAGAGCAGAAGTGGCAGTG	GGGAGGAGGGCTCCAGTC
Exon 8	CTGGGTGAGTTGACAGGTTG	ACTTCCCTCCTCCTCGGTC
Exon 9	AGTGTGGAAAGGAGGGGC	GATCCTGTTGCTTTGGTGG
Exon 10	CTAGGGACCTCTGGGGTAGC	GTGAGGGGCAGGAAAGC
Exon 11	ACAAGGGCTATGAGGGTGG	GCAAAGCCCTGAAGACAAAC
Exon 12	GCTCTGCAGGCTGTTCTACG	CTCAGCAGGACAGGGAGC
Exon 13	GTGTGAATTGTGGGGAAAGG	GATGGCAAGTGCACGACTC
Exon 14	CTGAGATCTGGAGTGCATGG	GTCCCTAGCATGGTTTGCTG
Exon 15	CCCCAGAGCCCAGTCTTG	AGGATGAGGGGAAGCCATC
Exon 16	CTGACTCGAAAGAGCCTGG	GGGAACAGAGCAGACCCTTG
Exon 17	ACCCTGGTCCTAGCTGGC	GGCAGGGGTACGGTTGTG
*PANK2*		
Exon 1A	GCTCTATTCCAGAGACCGAGTG	ATTTCCAACTTGAAATCTAACCAG
Exon 1B	ACCAGCCTGGACAACATAGTG	GACTGGAACAGAATTCAACTGAG
Exon 2	TTTCAGCACTTAGTTCACTTTAGG	CCAGAACTTCACCAATATAGCAC
Exon 3	TTATTAAGAGGACTGTGTGGAGTG	CTCATATTCCATGATCTTCCAGAC
Exon 4	TTTACTTCATGTGATGCCAGG	TCTTAAACAAACCACATTGTCTTC
Exon 5	GCACTGTACTTCTTCCATGAGG	CAGTCAGATGTCATACTCACCAAG
Exon 6	TTGTTGTAGATGATGCATACTTGG	AGAGTTTTAGGGACACAGGCAC
Exon 7	ACTGTTTAATGCAGGACGAATG	GATGACTACTCCAGCACAGACAAC
*FTL*		
Exon 4	GCCTCATTTCACACCTGTC	CTCCTCTTTCACTGGCATC

Oligonucleotide primers used to amplify the exonic regions and flanking introns of the phospholipase A2, group VI (*PLA2G6*), the pantothenate kinase 2 (*PANK2*) and the ferritin light chain (*FTL*) genes. Key: PCR, polymerase chain reaction.

**Table 2:** Clinical features in the cases with *PLA2G6* genetic mutations

Case	Age of onset	Age of death	First clinical symptom	Ataxia	Dystonia	Dysphagia	Other
1	18 years	36 years	Tripping and prone to falls	a	b	Late onset dysphagia requiring a gastrostomy at age 28	Rigidity and bradykinesia, emotional and aggressive outbursts, anarthria
2	22 years	32 years (alive)	Mood changes and aggression	c	a	c	Dystonia, rigidity and bradykinesia, emotional and aggressive outbursts
3	Infant	8 years	Developmental delay	Yes	b	a, gastrostomy	Bilateral optic atrophy. MRI − cerebellar hypoplasia with hypomyelination
4	Childhood	18 years	Dystonia and myoclonus	NA	d	a, gastrostomy	EEG right side slowing and spike slow wave discharges
5	14 months	8 years	Developmental delay	a	b	d	Hearing loss, seizures, cramps, flaccid tone. CT, EEG normal at 30 months
6	11 months	12 years (alive)	Psychomotor retardation	a	NA	a, G-tube inserted for feeding	Seizures, hypothermia, bradycardia, tracheostomy MRI shows iron deposition
7	Infantile	NA	Developmental delay	NA	NA	NA	NA

As with the PEG, the G-tube is use if patients require long-term tube feeding. Key: CT, computerized tomography; EEG, electroencephalogram; G, ; MRI, magnetic resonance imaging; NA, information not available; PEG, ; *PLA2G6*, phospholipase A2, Group VI; Yes, symptom present but no severity given.

a Severe symptom.

b Symptom not present.

c Mild symptom.

d Moderate symptom.

**Table 3:** *PLA2G6* gene mutations identified

Case	Sex	Ethnicity	Mutation	Amino acid change	Type and location	Diagnosis
1	F	British	c.109C>T c.1078 – 3C>A	p.R37X Splice site	Exon 2 Exon 8	Juvenile onset NAD
2	F	Greek	c.1715C>T	p.T572I	Exon 12 Homozygous	Adult onset NAD
3	M	American Caucasian	c.1061T>C c.1933C>T	p.L354P p.R654X	Exon 7 Exon 14	Infantile onset NAD
4	M	American Caucasian	c.319delC	p.L107FsX4	Exon 3 Heterozygous	Juvenile onset NAD
5	M	American	c.610 – 1G>T c.2370delTG	Splice site p.Y790X	Exon 5 Exon 17	Infantile onset NAD
6	M	Hispanic	c.1799G>A	p.R600Q	Exon 13 Homozygous	Infantile onset NAD
7	M	Portuguese	c.2370T>G	p.Y790X	Exon 17 Heterozygous	Infantile onset NAD

Key: AR, autosomal recessive; F, female; M, male; NAD, neuroaxonal dystrophy; *PLA2G6*, phospholipase A2, group VI.

**Table 4:** Pathological features in the cases with *PLA2G6* genetic mutations

Case	Amino acid change	Cortical atrophy	Cerebellar atrophy	Neuroaxonal spheroids	Lewy body pathology	Tau pathology
1 QSBB Brain and cord	p.R37X Splice site	No	Yes	a	a Braak stage 6; “diffuse neocortical Lewy body type pathology”	b Neurofibrillary tangles and neuropil threads
2^c^ QSBB Frontal cortical biopsy	p.T572I	N/A	N/A	d	a	e Neuropil threads
3 NICHD Brain	p.L354P p.R654X	N/A	N/A	e	b	Zero
4 NICHD Brain	p.L107FsX4	No	No	a	f Braak stage 6; “diffuse neocortical Lewy body type pathology”	a Neurofibrillary tangles and neuropil threads
5^g^ NICHD Brain and cord	Splice site p.Y790X	Yes	Yes	Yes	Yes^g^	Tau-positive glia^g^
6^h^ NICHD Sural nerve only	p.R600Q	N/A	N/A	Yes	N/A	N/A
7^h^ No tissue	p.Y790X	N/A	N/A	N/A	N/A	N/A

The sural nerve pathology was from the reporting hospital. Key: N/A, no information available; No, pathological feature not present; NICHD, case from National Institute of Child Health and Human Development, Brain and Tissue Bank, Baltimore, MD; *PLA2G6*, phospholipase A2, Group VI; QSBB, case from the Queen Square Brain Bank, London, UK; Yes, pathological feature present.

a Severity of pathological change severe.

b Severity of pathological change mild.

c Previously reported, formal assessment was performed in the present study (Tofaris et al., 2007).

d Severity of pathological change absent.

e Severity of pathological change moderate.

f Severity of pathological change very severe.

g Previously reported, no formal assessment in the present study as tissue was not available (Galvin et al., 2000).

h No brain tissue available.

**Table 5:** Sample of atypical cases with adult atypical NAD and parkinsonism show a greater number of missense and compound heterozygous mutations

Case	Mutation	Age of onset (years)	Phenotype	Neuropathology
Case 1 (here)	p.T572I	18	Dystonia-parkinsonism	Lewy bodies and tau
Case 2 (here)	p. R37X/c.1078–3C > A	22	Dystonia-parkinsonism	Lewy bodies and tau
Family 1	p.R741Q	10 and 26	Dystonia-parkinsonism	Not available
Family 2	p.R747W	18	Dystonia-parkinsonism	Not available
Iranian family	p.R632W	21, 22, and 25	Dystonia-parkinsonism	Not available

Key: NAD, neuroaxonal dystrophy.

## References

[bib1] Arawaka S., Saito Y., Murayama S., Mori H. (1998). Lewy body in neurodegeneration with brain iron accumulation type 1 is immunoreactive for alpha-synuclein. Neurology.

[bib2] Barbosa E.R., Bittar M.S., Bacheschi L.A., Comerlatti L.R., Scaff M. (1995). Precocious Parkinson's disease associated with “eye-of-the-tiger” type pallidal lesions [in Porguguese]. Arq. Neuropsiquiatr.

[bib3] Braak H., Del Tredici K., Rub U., de Vos R.A., Jansen Steur E.N., Braak E. (2003). Staging of brain pathology related to sporadic Parkinson's disease. Neurobiol. Aging.

[bib4] Curtis A.R., Fey C., Morris C.M., Bindoff L.A., Ince P.G., Chinnery P.F., Coulthard A., Jackson M.J., Jackson A.P., McHale D.P., Hay D., Barker W.A., Markham A.F., Bates D., Curtis A., Burn J. (2001). Mutation in the gene encoding ferritin light polypeptide causes dominant adult-onset basal ganglia disease. Nat. Genet.

[bib5] Duda J.E., Giasson B.I., Mabon M.E., Lee V.M., Trojanowski J.Q. (2002). Novel antibodies to synuclein show abundant striatal pathology in Lewy body diseases. Ann. Neurol.

[bib6] Galvin J.E., Giasson B., Hurtig H.I., Lee V.M., Trojanowski J.Q. (2000). Neurodegeneration with brain iron accumulation, type 1 is characterized by alpha-, beta-, and gamma-synuclein neuropathology. Am. J. Pathol.

[bib7] Gregory A., Polster B.J., Hayflick S.J. (2009). Clinical and genetic delineation of neurodegeneration with brain iron accumulation. J. Med. Genet.

[bib8] Gregory A., Westaway S.K., Holm I.E., Kotzbauer P.T., Hogarth P., Sonek S., Coryell J.C., Nguyen T.M., Nardocci N., Zorzi G., Rodriguez D., Desguerre I., Bertini E., Simonati A., Levinson B., Dias C., Barbot C., Carrilho I., Santos M., Malik I., Gitschier J., Hayflick S.J. (2008). Neurodegeneration associated with genetic defects in phospholipase A(2). Neurology.

[bib9] Guillerman R.P. (2000). The eye-of-the-tiger sign. Radiology.

[bib10] Hajek M., Adamovicova M., Herynek V., Skoch A., Jiru F., Krepelova A., Dezortova M. (2005). MR relaxometry and 1H MR spectroscopy for the determination of iron and metabolite concentrations in PKAN patients. Eur. Radiol.

[bib11] Hallervorden J., Spatz H. (1922). Eigenartige Erkrankung im cxtrapyramidalen System mit besonderer Beteiligung des globus pallidus nnd der Substantia nigra. Ein Beitrag zu den Beziehungen zwischen diesen beiden Zentren. Z. ges. Neurol. Psychiatr.

[bib12] Halliday G., Hely M., Reid W., Morris J. (2008). The progression of pathology in longitudinally followed patients with Parkinson's disease. Acta Neuropathol..

[bib13] Hayflick S., Westaway S. (2006). Pantothenate kinase 2 mutation without “eye-of-the-tiger” sign. Pediatr. Radiol.

[bib14] Hayflick S.J., Hartman M., Coryell J., Gitschier J., Rowley H. (2006). Brain MRI in neurodegeneration with brain iron accumulation with and without PANK2 mutations. AJNR Am. J. Neuroradiol.

[bib15] Hayflick S.J., Westaway S.K., Levinson B., Zhou B., Johnson M.A., Ching K.H., Gitschier J. (2003). Genetic, clinical, and radiographic delineation of Hallervorden–Spatz syndrome. N Engl J. Med.

[bib16] Hermann W., Reuter M., Barthel H., Dietrich J., Georgi P., Wagner A. (2000). Diagnosis of Hallervorden–Spatz disease using MRI, (one hundred and twenty-three) I-beta-CIT-SPECT and (one hundred and twenty-three) I-IBZM-SPECT. Eur. Neurol.

[bib17] Ishizawa T., Mattila P., Davies P., Wang D., Dickson D.W. (2003). Colocalization of tau and alpha-synuclein epitopes in Lewy bodies. J. Neuropathol. Exp. Neurol.

[bib18] Khateeb S., Flusser H., Ofir R., Shelef I., Narkis G., Vardi G., Shorer Z., Levy R., Galil A., Elbedour K., Birk O.S. (2006). PLA2G6 mutation underlies infantile neuroaxonal dystrophy. Am. J. Hum. Genet.

[bib19] Malik I., Turk J., Mancuso D.J., Montier L., Wohltmann M., Wozniak D.F., Schmidt R.E., Gross R.W., Kotzbauer P.T. (2008). Disrupted membrane homeostasis and accumulation of ubiquitinated proteins in a mouse model of infantile neuroaxonal dystrophy caused by PLA2G6 mutations. Am. J. Pathol.

[bib20] McKeith I.G., Dickson D.W., Lowe J., Emre M., O'Brien J.T., Feldman H., Cummings J., Duda J.E., Lippa C., Perry E.K., Aarsland D., Arai H., Ballard C.G., Boeve B., Burn D.J., Costa D., Del Ser T., Dubois B., Galasko D., Gauthier S., Goetz C.G., Gomez-Tortosa E., Halliday G., Hansen L.A., Hardy J., Iwatsubo T., Kalaria R.N., Kaufer D., Kenny R.A., Korczyn A., Kosaka K., Lee V.M., Lees A., Litvan I., Londos E., Lopez O.L., Minoshima S., Mizuno Y., Molina J.A., Mukaetova-Ladinska E.B., Pasquier F., Perry R.H., Schulz J.B., Trojanowski J.Q., Yamada M. (2005). Diagnosis and management of dementia with Lewy bodies: third report of the DLB Consortium. Neurology.

[bib21] McNeill A., Birchall D., Hayflick S.J., Gregory A., Schenk J.F., Zimmerman E.A., Shang H., Miyajima H., Chinnery P.F. (2008). T2* and FSE MRI distinguishes four subtypes of neurodegeneration with brain iron accumulation. Neurology.

[bib22] Morgan N.V., Westaway S.K., Morton J.E., Gregory A., Gissen P., Sonek S., Cangul H., Coryell J., Canham N., Nardocci N., Zorzi G., Pasha S., Rodriguez D., Desguerre I., Mubaidin A., Bertini E., Trembath R.C., Simonati A., Schanen C., Johnson C.A., Levinson B., Woods C.G., Wilmot B., Kramer P., Gitschier J., Maher E.R., Hayflick S.J. (2006). PLA2G6, encoding a phospholipase A2, is mutated in neurodegenerative disorders with high brain iron. Nat. Genet.

[bib23] Morita H., Inoue A., Yanagisawa N. (1992). A case of ceruloplasmin deficiency which showed dementia, ataxia and iron deposition in the brain. Rinsho Shinkeigaku.

[bib24] Neumann J., Bras J., Deas E., O'Sullivan S.S., Parkkinen L., Lachmann R.H., Li A., Holton J., Guerreiro R., Paudel R., Segarane B., Singleton A., Lees A., Hardy J., Houlden H., Revesz T., Wood N.W. (2009). Glucocerebrosidase mutations in clinical and pathologically proven Parkinson's disease. Brain.

[bib25] Neumann M., Adler S., Schluter O., Kremmer E., Benecke R., Kretzschmar H.A. (2000). Alpha-synuclein accumulation in a case of neurodegeneration with brain iron accumulation type 1 (NBIA-1, formerly Hallervorden–Spatz syndrome) with widespread cortical and brainstem-type Lewy bodies. Acta Neuropathol.

[bib26] Newell K.L., Boyer P., Gomez-Tortosa E., Hobbs W., Hedley-Whyte E.T., Vonsattel J.P., Hyman B.T. (1999). Alpha-synuclein immunoreactivity is present in axonal swellings in neuroaxonal dystrophy and acute traumatic brain injury. J. Neuropathol. Exp. Neurol.

[bib27] Odawara T., Iseki E., Yagishita S., Amano N., Kosaka K., Hasegawa K., Matsuda Y., Kowa H. (1992). An autopsied case of juvenile parkinsonism and dementia, with a widespread occurrence of Lewy bodies and spheroids. Clin. Neuropathol.

[bib28] Paisan-Ruiz C., Bhatia K.P., Li A., Hernandez D., Davis M., Wood N.W., Hardy J., Houlden H., Singleton A., Schneider S.A. (2009). Characterization of PLA2G6 as a locus for dystonia-parkinsonism. Ann. Neurol.

[bib29] Saito Y., Kawai M., Inoue K., Sasaki R., Arai H., Nanba E., Kuzuhara S., Ihara Y., Kanazawa I., Murayama S. (2000). Widespread expression of alpha-synuclein and tau immunoreactivity in Hallervorden–Spatz syndrome with protracted clinical course. J. Neurol. Sci.

[bib30] Seitelberger F. (1971). Neuropathological conditions related to neuroaxonal dystrophy. Acta Neuropathol.

[bib31] Sina F., Shojaee S., Elahi E., Paisan-Ruiz C. (2009). R632W mutation in PLA2G6 segregates with dystonia-parkinsonism in a consanguineous Iranian family. Eur. J. Neurol.

[bib32] Sugiyama H., Hainfellner J.A., Schmid-Siegel B., Budka H. (1993). Neuroaxonal dystrophy combined with diffuse Lewy body disease in a young adult. Clin. Neuropathol.

[bib33] Tofaris G.K., Revesz T., Jacques T.S., Papacostas S., Chataway J. (2007). Adult-onset neurodegeneration with brain iron accumulation and cortical alpha-synuclein and tau pathology: a distinct clinicopathological entity. Arch. Neurol.

[bib34] Tuite P.J., Provias J.P., Lang A.E. (1996). Atypical dopa responsive parkinsonism in a patient with megalencephaly, midbrain Lewy body disease, and some pathological features of Hallervorden–Spatz disease. J. Neurol. Neurosurg. Psychiatry.

[bib35] Wakabayashi K., Yoshimoto M., Fukushima T., Koide R., Horikawa Y., Morita T., Takahashi H. (1999). Widespread occurrence of alpha-synuclein/NACP-immunoreactive neuronal inclusions in juvenile and adult-onset Hallervorden–Spatz disease with Lewy bodies. Neuropathol. Appl. Neurobiol.

[bib36] Zhou B., Westaway S.K., Levinson B., Johnson M.A., Gitschier J., Hayflick S.J. (2001). A novel pantothenate kinase gene (PANK2) is defective in Hallervorden–Spatz syndrome. Nat. Genet.

